# Automated, in-vivo, whole-cell electrophysiology using an integrated patch-clamp amplifier

**DOI:** 10.1186/1471-2202-14-S1-P131

**Published:** 2013-07-08

**Authors:** Ilya Kolb, Gregory Holst, Brian Goldstein, Suhasa B Kodandaramaiah, Edward S Boyden, Eugenio Culurciello, Craig R Forest

**Affiliations:** 1 Wallace H. Coulter Department of Biomedical Engineering, Georgia Institute of Technology, Atlanta GA, 30332 USA; 2 George W. Woodruff School of Mechanical Engineering, Georgia Institute of Technology, Atlanta GA, 30332 USA; 3 Department of Electrical Engineering, Yale University, New Haven, CT 06520 USA; 4 Media Lab, Massachusetts Institute of Technology, Cambridge MA, 02139 USA; 5 McGovern Institute, Massachusetts Institute of Technology, Cambridge MA, 02139 USA; 6 Weldon School of Biomedical Engineering, Purdue University, West Lafayette, IN 47907 USA

## 

Whole-cell patch clamp recordings of neuronal activity *in vivo *exhibit signal quality sufficient to report synaptic and ion channel-mediated subthreshold events of importance for understanding neural processing during normal as well as drug-mediated behavior. Recently, we developed a prototype "autopatching" robot [1] that enables automated whole-cell patch clamp recording of neurons in a living mouse brain. While automation of the in-vivo patch clamping process opens up the possibility of multi-channel *in vivo *electrophysiology, scaling up the current system would dramatically increase system cost and complexity. Specifically, the current autopatching algorithm relies on several discrete pieces of hardware such as an amplifier, headstage and digitizer--as well as commercial software to communicate with the equipment and perform electrophysiology.

**Figure 1:**
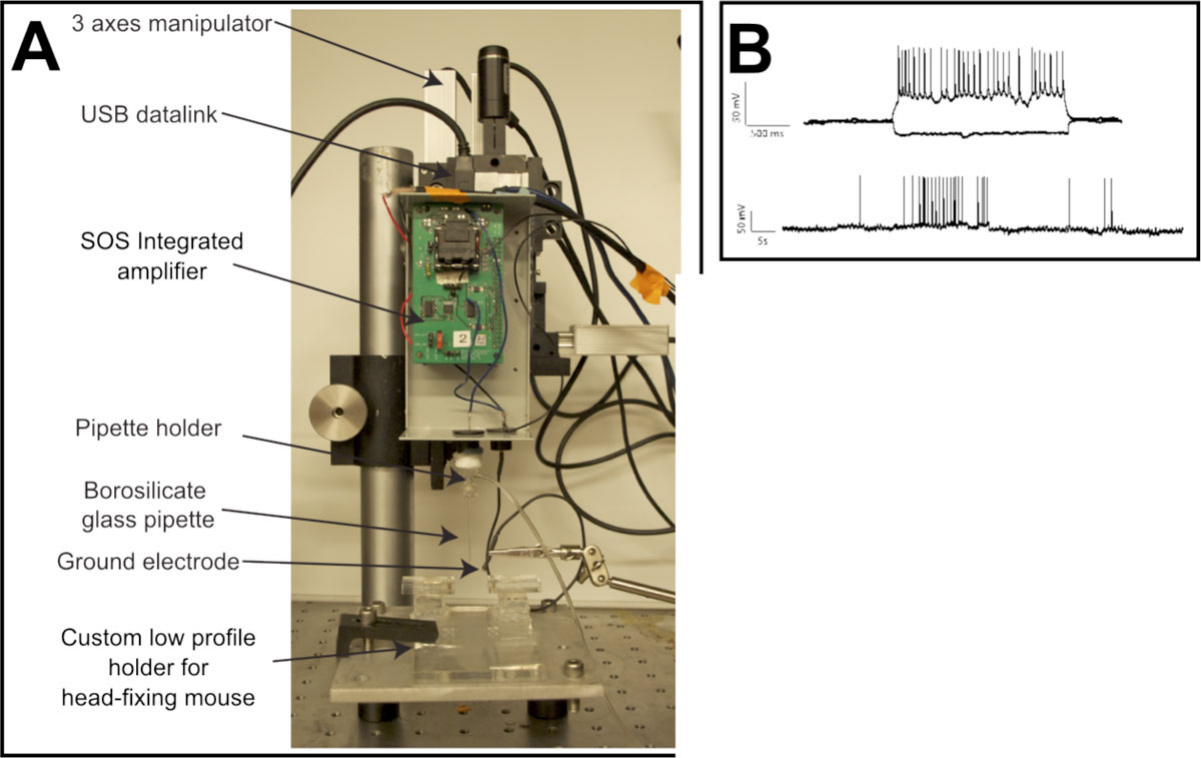
**(**A**) Single-channel patch chip amplifier in the patch clamping setup**. (**B**) Representative *in vivo *whole-cell recordings in current clamp mode using conventional pCLAMP software and the patch chip.

We will present an automated, in-vivo, whole cell electrophysiology suite that consists of a four-channel microchip-based patch clamp amplifier [2] and software that dramatically simplifies the setup necessary for traditional automated patch clamping. This system (Figure 1A) eliminates the need for the conventional amplifier, digitizer, headstage, and data acquisition board to effectively combine the high-throughput nature of the autopatcher [1] with the voltage and current clamp toolsets available in commercial software packages. Un-automated *in vivo *whole-cell recordings have already been demonstrated using a single-channel microchip-based patch clamp amplifier with conventional pCLAMP software (Molecular Devices, Sunnyvale, CA; Figure 1B). Preliminary results indicate that our simpler, multi-channel electrophysiology suite can perform computations required for automatic neuron detection, leading to successful whole-cell patches. We will report progress toward total automated integration and incorporation of electrophysiology tools into the software suite. The total cost required for the patch chip and the electrophysiology software suite should be several orders of magnitude less than that of conventional systems, and far simpler to handle, integrate with existing rigs, and use.
